# Can species distribution models really predict the expansion of invasive species?

**DOI:** 10.1371/journal.pone.0193085

**Published:** 2018-03-06

**Authors:** Morgane Barbet-Massin, Quentin Rome, Claire Villemant, Franck Courchamp

**Affiliations:** 1 Ecologie, Systématique et Evolution, Université Paris-Sud, CNRS, AgroParisTech, Université Paris-Saclay, Orsay, France; 2 ISYEB—UMR 7205 –CNRS, MNHN, UPMC, EPHE, Muséum national d’Histoire naturelle, Sorbonne Universités, Paris, France; 3 UMS 2006 Patrimoine Naturel–MNHN, AFB, CNRS, Muséum national d’Histoire naturelle, Paris, France; Universita degli Studi di Napoli Federico II, ITALY

## Abstract

Predictive studies are of paramount importance for biological invasions, one of the biggest threats for biodiversity. To help and better prioritize management strategies, species distribution models (SDMs) are often used to predict the potential invasive range of introduced species. Yet, SDMs have been regularly criticized, due to several strong limitations, such as violating the equilibrium assumption during the invasion process. Unfortunately, validation studies–with independent data–are too scarce to assess the predictive accuracy of SDMs in invasion biology. Yet, biological invasions allow to test SDMs usefulness, by retrospectively assessing whether they would have accurately predicted the latest ranges of invasion. Here, we assess the predictive accuracy of SDMs in predicting the expansion of invasive species. We used temporal occurrence data for the Asian hornet *Vespa velutina nigrithorax*, a species native to China that is invading Europe with a very fast rate. Specifically, we compared occurrence data from the last stage of invasion (independent validation points) to the climate suitability distribution predicted from models calibrated with data from the early stage of invasion. Despite the invasive species not being at equilibrium yet, the predicted climate suitability of validation points was high. SDMs can thus adequately predict the spread of *V*. *v*. *nigrithorax*, which appears to be—at least partially–climatically driven. In the case of *V*. *v*. *nigrithorax*, SDMs predictive accuracy was slightly but significantly better when models were calibrated with invasive data only, excluding native data. Although more validation studies for other invasion cases are needed to generalize our results, our findings are an important step towards validating the use of SDMs in invasion biology.

## Introduction

In the recent past, globalization has led to an increase of invasive species, a pattern likely to continue [1]. Besides being one of the biggest threat to biodiversity and ecosystems [2], biological invasions are also very costly to the global economy [3]. This increase of invasive species and their consequences on biodiversity and ecosystems raise numerous management and control issues [4,5]. Preventing an invasive species’ establishment and further spread is recognized as a more efficient and less costly management strategy than eradication, containment and control that may be required when the invasive species has fully established [6]. To that end, species distribution models (SDMs) are increasingly being used in invasion biology, especially to predict invasion risk [7–12] and optimize control strategies [13,14].

SDMs are also widely used in conservation biology, e.g. to predict the potential impact of climate change on genetic diversity [15,16], on species diversity [17–20] and on functional diversity [21,22] or to help reserve planning [23–25]. However, SDMs have been criticized for lacking mechanisms and independent validation, among other things [26]. Besides, two key assumptions of SDMs are often violated in invasion biology. First, niche conservatism is an assumption required for model transferability, whereby climate niches modeled with information from the native area are often projected onto new geographical spaces to estimate the likelihood of successful invasions there. In the context of invasion biology, niche conservatism differs from evolutionary niche conservatism (fundamental niche conserved over evolutionary time), as the question is to know whether the species realized niche is conserved over space. Yet, the assumption of niche conservatism over space is not always met, as the naturalized climatic niches of invasive species can differ from their natives climatic niches [27–29]. Second, until the latest stage of invasion, an invasive species is not yet at equilibrium with its environment [30], so its climatic niche is likely underestimated. Despite such criticisms, the important need of predictive models is such that SDMs are still often used in invasion biology. Indeed, validation studies (*i*.*e*., where SDM predictive accuracy is estimated with independent data and not through cross-validation only) being so scarce, SDMs have still not been fully proven to be inaccurate–or accurate -. A few pioneer studies aimed at assessing the predictive accuracy of SDMs in predicting species distribution changes with mixed results: a study showed that models had a good accuracy in predicting the range change of the Eurasian otter in Spain observed between two nation-wide surveys carried out ten years apart [31], whereas a study of deer species in Great Britain showed that SDMs predictions of range changes were no better than dispersal models [32]. Invasive species represent a good opportunity to evaluate SDMs predictive accuracy with independent data, as their invasive range can expand quickly. Indeed, invasive species whose invasion was closely monitored can be used to test whether records from the later stage of the invasion could have been predicted by a model calibrated with records only from the early stages of the invasion. Only very few studies have taken advantage of this opportunity to carry out validation studies with independent data [33–38], but they were carried out with simplistic envelop models or at small spatial scales. Besides, invasive species not being likely at equilibrium makes the use of common evaluation metrics (such as AUC, TSS…) not appropriate as observed absences can either be because of unsuitable environment or because the species did not disperse there yet [39]. More appropriate validation studies are thus very much needed.

*Vespa velutina nigrithorax*, or Asian hornet, is a perfect candidate species for such validation test. This insect native to China invaded France in 2004 [40] after its accidental introduction from China [41]. It spread rapidly, colonizing most of France at an approximate rate of 60–80 km per year [42,43] and progressively invaded other European countries: Spain in 2010, Portugal and Belgium in 2011, Italy in 2012, Germany in 2014 [44–48], the UK in 2016 [49] and finally the Netherlands where it was first recorded in 2017 (http://frelonasiatique.mnhn.fr/le-frelon-asiatique-detecte-aux-pays-bas/). The spread of this invasive species has been closely monitored since the species was introduced, so it is a perfect example for a validation study. In this study, after investigating whether *V*. *v*. *nigrithorax* is at equilibrium in its invasion range, we used more than 10,000 European invasion occurrences recorded between 2004 and 2015 to test whether occurrences from the later stage of invasion could have been predicted by models calibrated using occurrences from the earlier stage of invasion. We also took advantage of having independent validation data to test whether SDMs would have a better predictive accuracy of the ongoing invasive range if native data were accounted for, thereby responding to another strong question regarding the use of SDMs in invasion biology.

## Methods

### Presence data of *V*. *v*. *nigrithorax* in its native and invaded ranges

Presence data from the native Asian range was obtained by gathering information on museum specimens, published records and hornet sampling performed in China [50] (see S1 Fig in Supporting Information). As for the invaded range in Europe, the species was mostly observed in France, where it was first seen in 2004. Data from the French part of the invaded range came from the INPN database that aggregates all validated French records (https://inpn.mnhn.fr/), including nests but also presences of workers in regions where nests have not been found yet. To this French database, we added the recent locations reported in other European countries (Spain, Portugal, Italy, Belgium and Germany) [44–48,51]. Overall, we obtained 10,395 records in the European invaded range observed between 2004 and 2015.

### Climate data

We used the same eight climatic variables as in previous studies for the niche modelling of *V*. *v*. *nigrithorax* [50,52]. We considered: (1) annual mean temperature, (2) mean temperature of the warmest month, (3) mean temperature of the coldest month, (4) temperature seasonality, (5) annual precipitation, (6) precipitation of the wettest month, (7) precipitation of the driest month and (8) precipitation seasonality. The seasonality is the coefficient of variation of the monthly means. Current data were downloaded from the worldclim database [53] (http://www.worldclim.org/) as 2.5 arc-min grids (subset of the 19 bioclim variables). These data are interpolations from observed data representative of current climatic conditions.

### Is *V. v. nigrithorax* at equilibrium in its invaded range?

SDMs are often criticized when used in invasion biology because the equilibrium assumption is often violated [30]. Therefore, we investigated whether *V*. *v*. *nigrithorax* is at equilibrium in its invaded range by comparing the climatic niche occupied by the species during the first span of its invasion (2004–2010) to the one occupied now (2011–2015 invasion data). After being first observed in 2004, *V*. *v*. *nigrithorax* was observed twice in 2005 before really starting its spread in 2006 (more than 100 records over seven departments). Thus, the split of occurrence data into earlier and later stage of invasion in 2010 represents an equal 5-year length of geographical spread for each invasion stage. Visualization of the climatic niche and tests of niche equivalency and niche similarity were realized following the methods described by Broennimann et al. [54,55]. The first step consists of calculating the density of occurrences along the first two axes of a climate PCA (with the same variables used for the SDMs). The niche overlap was then calculated and niche equivalency and similarity were statistically tested [54].

### Climate suitability modeling

Climate suitability of *V*. *v*. *nigrithorax* was modeled by running eight different modeling techniques implemented within the *biomod2* package (3. 3–7 version) [56] in R [57]: three regression methods (GLM, GAM and MARS), two classification methods (CTA and FDA) and three machine learning methods (ANN, BRT and RF). We built two sets of models: one that accounted for presence data from the invaded range only and one that accounted for presence data from both the native and the invaded range. In order to evaluate whether the ongoing invasion in Europe can be predicted by climate suitability modeling, we used only presence data from the earlier stage of the invasion (2004–2010), so that records from the later stage of the invasion could be used as evaluation data. As no absence data were available for the species, pseudo-absences were randomly drawn [58]. For models using presence data from the invaded range only, pseudo-absences were chosen in Europe, whereas for models using presence data from both the invaded and the native range, pseudo-absences were chosen in the South-East part of Asia and in Europe. In both cases, we used 10,000 random pseudo-absences, with the total weight of presences being equal to the total weight of pseudo-absences [58]. As results might depend on the choice of pseudo-absences, models were replicated three times (with different pseudo-absences selection) [58]. To obtain a consensus distribution, we used an ensemble forecast technique [59]: the consensus distribution was calculated as the average of all distributions across modeling techniques and pseudo-absences replicates.

Model predictive accuracy was evaluated by assessing how well data recorded during the later stage of the invasion (2011–2015, hereafter called evaluation data) was predicted by the models that were calibrated with data from the early stage of the invasion only (2004–2010, hereafter called calibration data). To this end, we extracted the predicted climate suitability values for all invasive records from the evaluation data. Although the species displays one of the fastest invasive rate, with founder queens in flight mill experiments able to travel over 40 km a day [42] or fly an average of 18 km per day covering up to 200 km over 10 days [43], dispersal (in all cardinal directions) remains likely to be a limiting factor to the natural spreading. *V*. *v*. *nigrithorax* is therefore more likely to colonize areas close to where it first invaded. Thus, we need to compare the predicted climate suitability values of evaluation data to random points being within the same distance to the first invasion data. A better predictive accuracy is obtained when the climate suitability of validation point is higher than expected (given its distance to the first invasion record in this case). For a given validation point, we can thus compare its predicted climate suitability to the distribution of climate suitability values of all points being at the same distance from the first invasion record. That way, we can infer in which percentile of the distribution the validation point falls. A better predictive accuracy is obtained when percentiles of validation points are higher.

As results might depend on the cut-off year chosen to split the invasive data into calibration data and evaluation data, sensitivity analyses were carried out by applying different cut-off years. With that in mind, all analyses (SDM calibration and SDM evaluation) were carried out nine times, with cut-off years going from 2006 to 2014.

## Results

**Fig 1:**
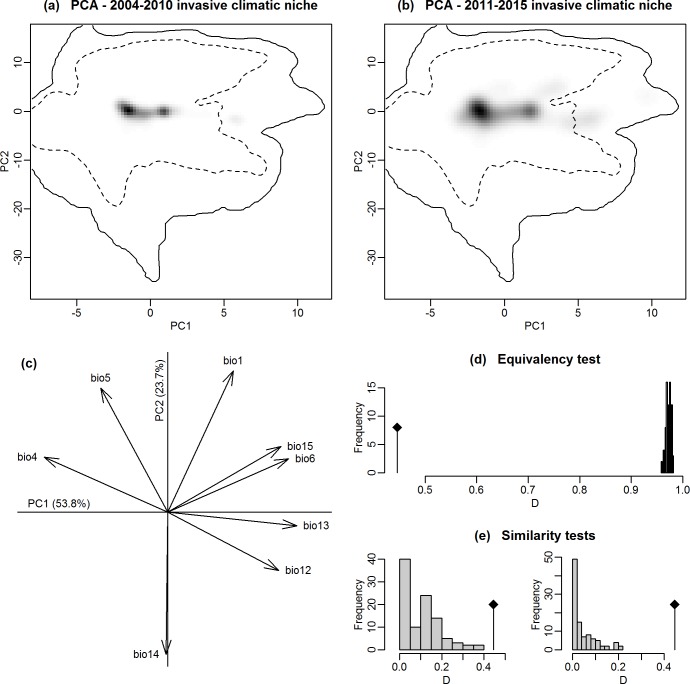
Is *V*. *v*. *nigrithorax* niche at equilibrium in its invaded range? Climatic niche occupied by *V*. *v*. *nigrithorax* in its European invasion range during 2004–2010 (a) and 2011–2015 (b) along the first two axes of the PCA (see (c) for details), showing an evolution during the two periods. Grey shading depicts the occurrence density of the species. The solid and dashed contour lines represent 100% and 50% respectively of the available (background) climate in Europe. (c) Contribution of the climate variables to the first two axes of the PCA (bio1: annual mean temperature, bio4: temperature seasonality, bio5: mean temperature of the warmest month, bio6: mean temperature of the coldest month, bio12: annual precipitation, bio13: precipitation of the wettest month, bio14: precipitation of the driest month, and bio15: precipitation seasonality). (d) Histograms showing the observed niche overlap D (D = 0.45) (bars with a diamond) and simulated niche overlaps (grey bars) on which tests of niche equivalency and niche similarity were calculated from 1000 iterations [54]: niches are similar but not equivalent.

The climatic niche occupied by *V*. *v*. *nigrithorax* in its invasion range clearly extended during the past few years (Fig 1A and 1B), as there is only a 45% overlap between the climatic niche occupied between 2004 and 2010 and the climatic niche occupied between 2011 and 2015. Statistical tests show that both niches are similar but not equivalent (Fig 1D and 1E). As the climatic niche first occupied by the species in its invasion range is still occupied, we could have expected both niches to be similar. Both niches not being equivalent further shows that part of the climatic niche occupied by the species between 2011 and 2015 was not occupied between 2004 and 2010. This means that in 2010 the species was not yet at equilibrium with its environment in Europe.

**Fig 2:**
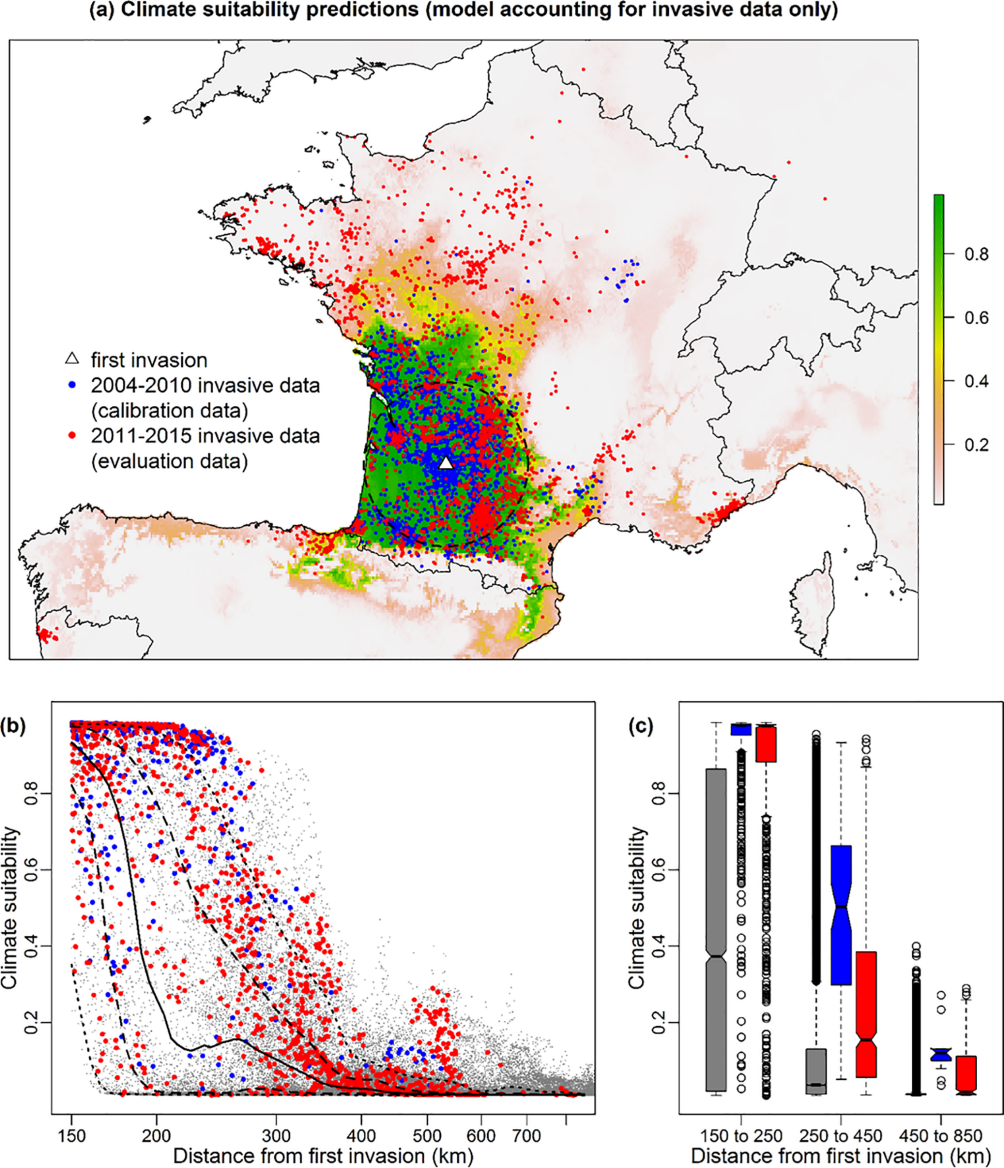
SDMs predictions and predictive accuracy. (a) Climate suitability gradient map, from 0 to 1, predicted by the model (current ensemble consensus) using invasive data from 2004 to 2010 (blue points). Red points represent invasive data recorded after 2010 (2011–2015) that are used to evaluate the model. The dotted circle around the first invasion data (blue triangle) delimits all points that are within 150km of the first invasion data. (b) Climate suitability of all possible points (between 150 and 850 km of the first invasion data) according to their distance to the first invasion (grey points). The full line represents the median climate suitability according to the distance, whereas the dotted lines represent the 10%, 30%, 70% and 90% quantiles (blue and red points as above). Evaluation (red) points above the median have a higher predicted suitability than expected given their distance to the first invasion occurrence. (c) Boxplots representing the range of climate suitability values for all possible points (grey) and invasive data (calibration data in blue and evaluation data in red) depending on their distance to the first invasion data. In all three cases, the predicted suitability of evaluation points is lower than the predicted suitability of calibration points, but is higher than expected given their distance to the first invasion occurrence (all possible points, in grey).

Climate suitability predictions differ depending on whether native data were accounted for (Fig 2 & S2 Fig). When accounting only for invasion data, climate suitability is high mainly in the southwestern part of France, where the invasion initiated (Fig 2A). However, when we accounted for both native and invasive data, high climate suitability is further predicted in the north of France, in Belgium, in northern Italy and in northern Spain (S3A Fig). In both cases, the predicted climate suitability of evaluation points (2011–2015, or “late invasion” data) is higher than expected by chance given their distance to the first invasion data (Fig 2 & S2 Fig). Indeed, out of the 2,534 evaluation points that are further away than 150 km from the first invasion record, more than 60% of them have their predicted climate suitability above the 70^th^ percentile of all background points within the same distance, whereas less than 3% of the evaluation points have their predicted climate suitability below the 30^th^ percentile (Fig 2 & S2 Fig). Besides, although the predicted suitability of the evaluation points that are the further away (further than 450 km of the first invasion occurrence, but within 850 km) is lower than for closer evaluation points (Fig 2 & S2 Fig), it is still higher than expected given their distance. Indeed, in both cases, more evaluation points have their predicted climate suitability above the 70^th^ percentile of all background points within the same distance of the first invasion occurrence (31% vs 56% with or without accounting for native data), than below the 30^th^ percentile (0% in both cases) (Fig 2 & S2 Fig).

Results do not depend on the cut-off year that was used to split the data into calibration data and evaluation data, as results were very similar for the other cut-off years that were tested (S3 & S4 Figs). Such overall results thus mean that climate influences–at least to some extent—the ongoing invasion of *V*. *v*. *nigrithorax* in Europe and that this influence can be predicted by SDMs, despite the species not being at equilibrium yet.

**Fig 3:**
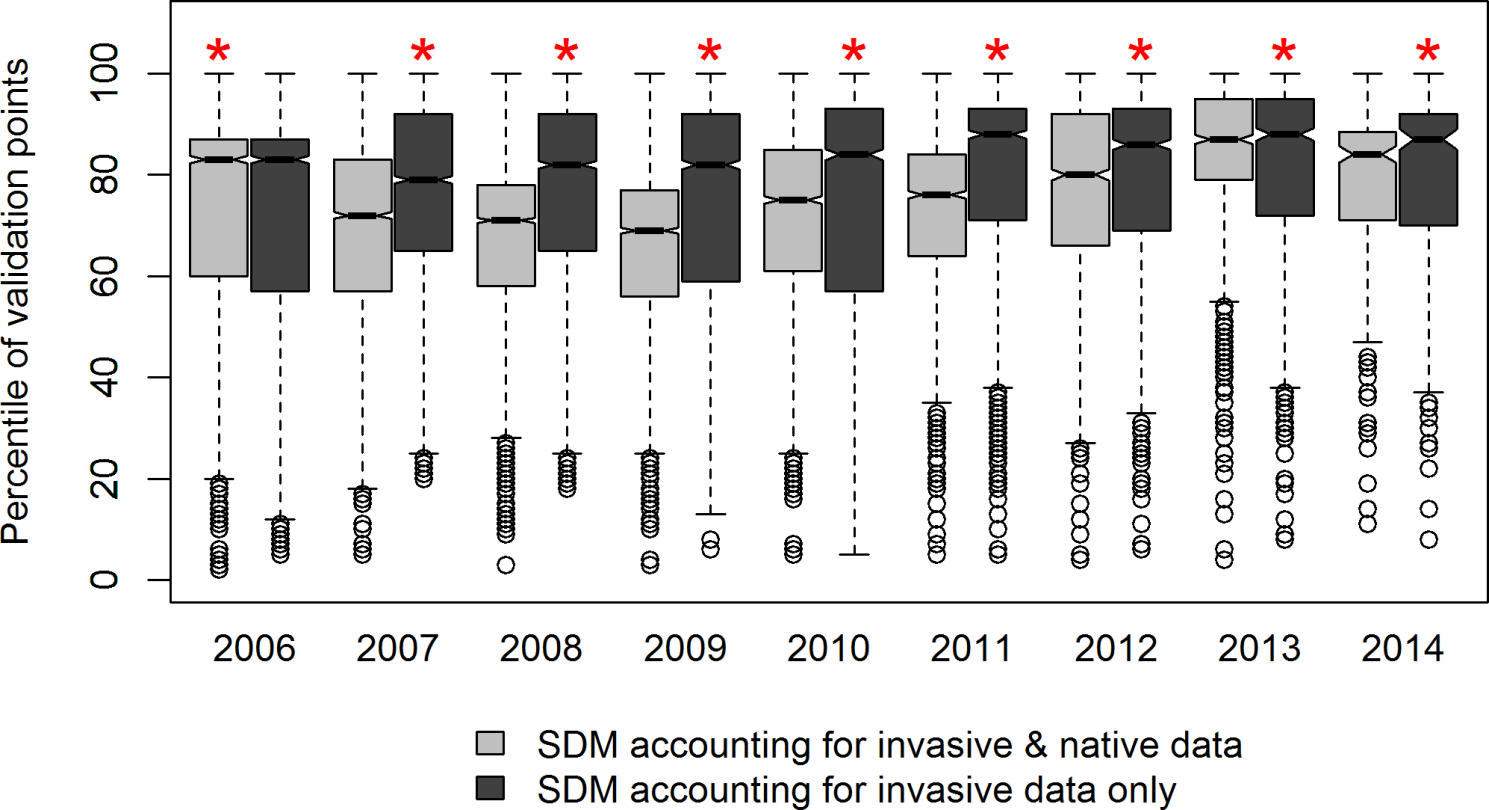
Comparing SDMs predictive accuracy when trained with or without native data. Percentiles of validation points (further than 150km from the first invasion record) depending on whether or not native data was accounted for to calibrate the models and on the cut-off year that was used to split the invasive data into calibration and evaluation data. Percentiles are obtained by comparing the predicted climate suitability of a given validation point to the distribution of climate suitability values of all points being at the same distance from the first invasion record than the validation point (i.e., grey points in Fig 2B). Percentiles higher than 50^th^ thus mean that the predicted climate suitability of the validation point is higher than expected given its distance to the first invasion record. For all cut-off years, paired t-test were computed to assess the difference between models with and without native data: a red star indicates significantly higher values (S1 Table).

Furthermore, even though modeling methods provide good predictions, the predictions still differ according to whether or not native records were taken into account (Fig 2 & S2 Fig). We can thus further investigate whether one option provides more accurate results than the other. In our case study, percentiles of validation points were significantly higher when the climate suitability was predicted by models accounting for invasive data only (for all cut-off years, except for 2006, opposite result) (Fig 3 & S1 Table). Overall, models thus seem to have a better predictive accuracy when accounting for invasive data only.

## Discussion

Using the unique features of an invasion closely monitored in space and time, we demonstrated that despite some known limitations, SDMs can be a powerful tool to predict where invasive species will spread next. In fact, our case study does show that *V*. *v*. *nigrithorax* is not at equilibrium with its environment in its European invaded ranges (Fig 1). This finding is consistent with studies focusing on other invasive species [30,36]. The equilibrium hypothesis being an important assumption, its violation needs to be acknowledged when interpreting SDMs predictions [26]. Indeed, violating the equilibrium hypothesis has some consequences when modeling species distributions, among which underestimating the potential climatic niche of the species, which can in turn lead to underestimating the geographical area the species can invade [36]. However, predicting the full potential invasive range of an invasive species may not be as relevant as accurately predicting the areas that are more likely to be colonized next. Indeed, given the cost of species monitoring and surveillance for the early detection of invasive species, it is more relevant to predict areas that might be invaded next rather than all potential areas that could be reached by the invader if the species achieved its climate equilibrium. Information regarding the areas that might be invaded next could indeed be used by managers for a cost-effective effort on monitoring and controlling such areas. For example, in the case of *V*. *v*. *nigrithorax*, whose invasion can be most efficiently controlled by an early detection followed by nest removal [43,60], monitoring efforts need to be implemented within the highest suitable areas within the already invaded range, as well as within the highest suitable areas that are the closest to the already invaded range. Improved detection techniques would further increase the efficiency and decrease the costs of monitoring/controlling the invasion [61]. Therefore, even if invasive species distribution models cannot predict the full potential invasion range of an invasive species that has just established [36,62], they can still be very valuable for invasive species management. Yet, validation is needed for model reliability and credibility, especially when management decisions are based upon it [63].

Here, we showed that models calibrated with data from the earlier stage of invasion predicted adequately the recent invasive data of *V*. *v*. *nigrithorax*: new invasion observations had higher climatic suitability than expected from their distance to the first invasion occurrence. Although invasive species present a good opportunity to test SDMs predictions with independent data, this has rarely been done so far for the ongoing range expansions of invasive species. The few studies that pioneered this approach used evaluation metrics that have since been shown not to be appropriate when the species is not at equilibrium. Previous studies also showed that SDMs can be used to predict invasions under climate change, through validation from field data [64], as the performance in the field of three plant species was highly correlated with SDMs predicted climate suitability. Although further studies will be needed with other species, our study indicates that SDM can be used in invasion biology to better predict where the species is most likely to spread next, once an invasion has started. It is thus very important to monitor invasive species from the start of the invasion, so as to gather a large enough amount of information to run predictive SDMs. Besides, here we only considered climate variables, but model predictions can likely be improved by also considering non climatic drivers such as land-use variables [65], since predictions depend on the variables used to compute the models [66,67].

Numerous studies on invasive species distribution advocated to use distribution data from both the native and the invasive range [68,69]. In fact, if the species climatic niche is conserved from its native range to its invaded range, distribution data from the native range can be very valuable to characterize the full potential climatic niche of the species and thus the full geographical space it can invade. In this context, SDMs calibrated with distribution data from only the invasive range might under-predict the potential invasive range if the species is not at equilibrium yet [36]. However, if niche conservatism during invasions has been shown for some species [70,71], other studies revealed niche shifts during invasions [29,72,73], highlighting an inconsistent pattern of niche conservatism during invasions [74]. Accounting for native distribution data when calibrating invasive SDM may thus not improve their predictive accuracy in all cases. Here, we took advantage of having independent validation data to investigate this issue. In the case of *V*. *v*. *nigrithorax*, the invasive range is clearly predicted to be larger when accounting for native data (Fig 2 & S2 Fig). It is thus clear that at this time of the invasion, the climatic niche occupied by *V*. *v*. *nigrithorax* in its invaded range differs from the one occupied in its native range. If the native climatic niche is to be conserved, the full potential invasive range of the species might be better predicted by accounting for both invasive and native data, as the species is not at equilibrium with its environment in its invasive range yet (Fig 1). However, if the native climatic niche of *V*. *v*. *nigrithorax* is not to be conserved, accounting for native data when modeling its potential invasive range might lead to overprediction. Furthermore, predicting the full potential invasive range might not be as relevant as predicting the areas that are most likely to be invaded next, from a management point of view. Actually, the model predictive accuracy is slightly but significantly better when accounting for invasive data only (Fig 3). Thus, if the modeling purpose is to predict which areas the species is most likely to invade next, it is better to perform the SDMs without accounting for native data. Of course, similar studies need to be carried out for other species to know whether we can generalize such results or whether it depends on the species (as it appears to be the case for niche conservatism during invasions). Furthermore, although significant, the difference in model predictive accuracy is slight (Fig 3 & S1 Table), highlighting a very good predictive accuracy even when performing SDMs with both native and invasive data.

## Conclusions

SDMs are increasingly used in ecology whether to predict the potential impact of global change or to predict the potential invasive range of introduced species. Yet, they are often criticized, especially because their predictive accuracy cannot be truly estimated due to a lack of independent validation data. Our study of the invasion of *V*. *v*. *nigrithorax* showed that the predicted climate suitability of independent validation points was very good. Such a result means that the spread of *V*. *v*. *nigrithorax* is–at least partially–climatically driven and can be accurately predicted by SDMs. In the case of *V*. *v*. *nigrithorax*, SDMs predictive accuracy was slightly but significantly better when models were calibrated with invasive data only, excluding native data. Although more validation studies for other cases of alien invasion are needed to generalize our results, our findings validate the use of SDMs in invasion biology.

## Supporting information

S1 Table Comparing SDMs predictive accuracy for models trained with or without native data. (DOCX)

S1 Fig Native data. (DOCX)

S2 Fig SDMs predictions and predictive accuracy accounting for both native data and invasive data. (DOCX)

S3 Fig SDMs (trained with invasive data only) predictive accuracy for all cutoff years. (DOCX)

S4 Fig SDMs (trained with invasive and native data) predictive accuracy for all cutoff years. (DOCX)

## References

[1] Seebens H, Blackburn TM, Dyer EE, Genovesi P, Hulme PE, Jeschke JM, et al No saturation in the accumulation of alien species worldwide. Nat Commun. 2017;8: 14435 doi: 10.1038/ncomms14435 28198420 10.1038/ncomms14435 PMC5316856

[2] Bellard C, Cassey P, Blackburn TM. Alien species as a driver of recent extinctions. Biol Lett. 2016;12: 20150623 doi: 10.1098/rsbl.2015.0623 26888913 10.1098/rsbl.2015.0623 PMC4780541

[3] Bradshaw CJA, Leroy B, Bellard C, Roiz D, Albert C, Fournier A, et al Massive yet grossly underestimated global costs of invasive insects. Nat Commun. 2016;7: 12986 doi: 10.1038/ncomms12986 27698460 10.1038/ncomms12986 PMC5059451

[4] Lodge DM, Williams S, MacIsaac HJ, Hayes KR, Leung B, Reichard S, et al Biological invasions: Recommendations for US policy and management. Ecol Appl. 2006;16: 2035–2054. doi: 10.1890/1051-0761(2006)016[2035:BIRFUP]2.0.CO;2 17205888 10.1890/1051-0761(2006)016[2035:birfup]2.0.co;2

[5] Hulme PE. Trade, transport and trouble: managing invasive species pathways in an era of globalization. J Appl Ecol. 2009;46: 10–18. doi: 10.1111/j.1365-2664.2008.01600.x

[6] Simberloff D, Martin J-L, Genovesi P, Maris V, Wardle DA, Aronson J, et al Impacts of biological invasions: what’s what and the way forward. Trends Ecol Evol. 2013;28: 58–66. doi: 10.1016/j.tree.2012.07.013 22889499 10.1016/j.tree.2012.07.013

[7] Bradley BA, Wilcove DS, Oppenheimer M. Climate change increases risk of plant invasion in the Eastern United States. Biol Invasions. 2010;12: 1855–1872. doi: 10.1007/s10530-009-9597-y

[8] Jiménez-Valverde A, Peterson AT, Soberón J, Overton JM, Aragón P, Lobo JM. Use of niche models in invasive species risk assessments. Biol Invasions. 2011;13: 2785–2797. doi: 10.1007/s10530-011-9963-4

[9] Lecocq T, Rasmont P, Harpke A, Schweiger O. Improving International Trade Regulation by Considering Intraspecific Variation for Invasion Risk Assessment of Commercially Traded Species: The Bombus terrestris Case. Conserv Lett. 2016;9: 281–289. doi: 10.1111/conl.12215

[10] Kramer AM, Annis G, Wittmann ME, Chadderton WL, Rutherford ES, Lodge DM, et al Suitability of Laurentian Great Lakes for invasive species based on global species distribution models and local habitat. Ecosphere. 2017;8: e01883 doi: 10.1002/ecs2.1883

[11] Bosso L, Conno CD, Russo D. Modelling the Risk Posed by the Zebra Mussel Dreissena polymorpha: Italy as a Case Study. Environ Manage. 2017;60: 304–313. doi: 10.1007/s00267-017-0882-8 28493016 10.1007/s00267-017-0882-8

[12] Tingley R, García-Díaz P, Arantes CRR, Cassey P. Integrating transport pressure data and species distribution models to estimate invasion risk for alien stowaways. Ecography. 2017; n/a-n/a. doi: 10.1111/ecog.02841

[13] Giljohann KM, Hauser CE, Williams NSG, Moore JL. Optimizing invasive species control across space: willow invasion management in the Australian Alps. J Appl Ecol. 2011;48: 1286–1294. doi: 10.1111/j.1365-2664.2011.02016.x

[14] Tulloch AIT, Tulloch VJD, Evans MC, Mills M. The Value of Using Feasibility Models in Systematic Conservation Planning to Predict Landholder Management Uptake. Conserv Biol. 2014;28: 1462–1473. doi: 10.1111/cobi.12403 25382827 10.1111/cobi.12403

[15] Alsos IG, Ehrich D, Thuiller W, Eidesen PB, Tribsch A, Schonswetter P, et al Genetic consequences of climate change for northern plants. Proc R Soc B-Biol Sci. 2012;279: 2042–2051. doi: 10.1098/rspb.2011.2363 22217725 10.1098/rspb.2011.2363 PMC3311896

[16] Pauls SU, Nowak C, Balint M, Pfenninger M. The impact of global climate change on genetic diversity within populations and species. Mol Ecol. 2013;22: 925–946. doi: 10.1111/mec.12152 23279006 10.1111/mec.12152

[17] Maiorano L, Falcucci A, Zimmermann NE, Psomas A, Pottier J, Baisero D, et al The future of terrestrial mammals in the Mediterranean basin under climate change. Philos Trans R Soc B-Biol Sci. 2011;366: 2681–2692. doi: 10.1098/rstb.2011.0121 21844047 10.1098/rstb.2011.0121 PMC3140741

[18] Barbet-Massin M, Thuiller W, Jiguet F. The fate of European breeding birds under climate, land-use and dispersal scenarios. Glob Change Biol. 2012;18: 881–890. doi: 10.1111/j.1365-2486.2011.02552.x

[19] Garcia RA, Burgess ND, Cabeza M, Rahbek C, Araújo MB. Exploring consensus in 21st century projections of climatically suitable areas for African vertebrates. Glob Change Biol. 2012;18: 1253–1269. doi: 10.1111/j.1365-2486.2011.02605.x

[20] Domisch S, Araújo MB, Bonada N, Pauls SU, Jähnig SC, Haase P. Modelling distribution in European stream macroinvertebrates under future climates. Glob Change Biol. 2013;19: 752–762. doi: 10.1111/gcb.12107 23504833 10.1111/gcb.12107

[21] Thuiller W, Pironon S, Psomas A, Barbet-Massin M, Jiguet F, Lavergne S, et al The European functional tree of bird life in the face of global change. Nat Commun. 2014;5 doi: 10.1038/ncomms4118 24452245 10.1038/ncomms4118 PMC3999515

[22] Barbet-Massin M, Jetz W. The effect of range changes on the functional turnover, structure and diversity of bird assemblages under future climate scenarios. Glob Change Biol. 2015;21: 2917–2928. doi: 10.1111/gcb.12905 25931153 10.1111/gcb.12905

[23] Hannah L, Midgley G, Andelman S, Araújo M, Hughes G, Martinez-Meyer E, et al Protected area needs in a changing climate. Front Ecol Environ. 2007;5: 131–138. doi: 10.1890/1540-9295(2007)5[131:PANIAC]2.0.CO;2

[24] Marini MÂ, Barbet-Massin M, Lopes LE, Jiguet F. Major current and future gaps of Brazilian reserves to protect Neotropical savanna birds. Biol Conserv. 2009;142: 3039–3050. doi: 10.1016/j.biocon.2009.08.002

[25] Guisan A, Tingley R, Baumgartner JB, Naujokaitis-Lewis I, Sutcliffe PR, Tulloch AIT, et al Predicting species distributions for conservation decisions. Ecol Lett. 2013;16: 1424–1435. doi: 10.1111/ele.12189 24134332 10.1111/ele.12189 PMC4280402

[26] Araújo MB, Peterson AT. Uses and misuses of bioclimatic envelope modeling. Ecology. 2012;93: 1527–1539. doi: 10.1890/11-1930.1 22919900 10.1890/11-1930.1

[27] Medley KA. Niche shifts during the global invasion of the Asian tiger mosquito, Aedes albopictus Skuse (Culicidae), revealed by reciprocal distribution models. Glob Ecol Biogeogr. 2010;19: 122–133. doi: 10.1111/j.1466-8238.2009.00497.x

[28] Early R, Sax DF. Climatic niche shifts between species’ native and naturalized ranges raise concern for ecological forecasts during invasions and climate change. Glob Ecol Biogeogr. 2014;23: 1356–1365. doi: 10.1111/geb.12208

[29] Parravicini V, Azzurro E, Kulbicki M, Belmaker J. Niche shift can impair the ability to predict invasion risk in the marine realm: an illustration using Mediterranean fish invaders. Ecol Lett. 2015;18: 246–253. doi: 10.1111/ele.12401 25626355 10.1111/ele.12401

[30] Gallien L, Douzet R, Pratte S, Zimmermann NE, Thuiller W. Invasive species distribution models–how violating the equilibrium assumption can create new insights. Glob Ecol Biogeogr. 2012;21: 1126–1136. doi: 10.1111/j.1466-8238.2012.00768.x

[31] Areias-Guerreiro J, Mira A, Marcia Barbosa A. How well can models predict changes in species distributions? A 13-year-old otter model revisited. Hystrix-Ital J Mammal. 2016;27 doi: 10.4404/hystrix-27.1–11867

[32] Rodriguez-Rey M, Jimenez-Valverde A, Acevedo P. Species distribution models predict range expansion better than chance but not better than a simple dispersal model. Ecol Model. 2013;256: 1–5. doi: 10.1016/j.ecolmodel.2013.01.024

[33] Loo SE, Mac Nally R, Lake PS. Forecasting New Zealand mudsnail invasion range: Model comparisons using native and invaded ranges. Ecol Appl. 2007;17: 181–189. doi: 10.1890/1051-0761(2007)017[0181:FNZMIR]2.0.CO;2 17479844 10.1890/1051-0761(2007)017[0181:fnzmir]2.0.co;2

[34] Jarnevich CS, Holcombe TR, Barnett DT, Stohlgren TJ, Kartesz JT. Forecasting Weed Distributions using Climate Data: A GIS Early Warning Tool. Invasive Plant Sci Manag. 2010;3: 365–375. doi: 10.1614/IPSM-08-073.1

[35] Jones CC. Challenges in predicting the future distributions of invasive plant species. For Ecol Manag. 2012;284: 69–77. doi: 10.1016/j.foreco.2012.07.024

[36] Vaclavik T, Meentemeyer RK. Equilibrium or not? Modelling potential distribution of invasive species in different stages of invasion. Divers Distrib. 2012;18: 73–83. doi: 10.1111/j.1472-4642.2011.00854.x

[37] Crall AW, Jarnevich CS, Panke B, Young N, Renz M, Morisette J. Using habitat suitability models to target invasive plant species surveys. Ecol Appl. 2013;23: 60–72. doi: 10.1890/12-0465.1 23495636 10.1890/12-0465.1

[38] West AM, Kumar S, Brown CS, Stohlgren TJ, Bromberg J. Field validation of an invasive species Maxent model. Ecol Inform. 2016;36: 126–134. doi: 10.1016/j.ecoinf.2016.11.001

[39] Hattab T, Garzón-López CX, Ewald M, Skowronek S, Aerts R, Horen H, et al A unified framework to model the potential and realized distributions of invasive species within the invaded range. Divers Distrib. 2017;23: 806–819. doi: 10.1111/ddi.12566

[40] Haxaire J, Bouguet J, Tamisier J. Vespa velutina Lepeletier, 1836, a formidable addition for French fauna (Hym., Vespidae). Bull Société Entomol Fr. 2006;111: 194.

[41] Arca M, Mougel F, Guillemaud T, Dupas S, Rome Q, Perrard A, et al Reconstructing the invasion and the demographic history of the yellow-legged hornet, Vespa velutina, in Europe. Biol Invasions. 2015;17: 2357–2371. doi: 10.1007/s10530-015-0880-9

[42] Rome Q, Muller FJ, Touret-Alby A, Darrouzet E, Perrard A, Villemant C. Caste differentiation and seasonal changes in Vespa velutina (Hym.: Vespidae) colonies in its introduced range. J Appl Entomol. 2015;139: 771–782. doi: 10.1111/jen.12210

[43] Robinet C, Suppo C, Darrouzet E. Rapid spread of the invasive yellow-legged hornet in France: the role of human-mediated dispersal and the effects of control measures. J Appl Ecol. 2016; n/a-n/a. doi: 10.1111/1365-2664.12724

[44] López S, González M, Goldarazena A. Vespa velutina Lepeletier, 1836 (Hymenoptera: Vespidae): first records in Iberian Peninsula. EPPO Bull. 2011;41: 439–441. doi: 10.1111/j.1365-2338.2011.02513.x

[45] Porporato M, Manino A, Laurino D, Demichelis S. Vespa velutina Lepeletier (Hymenoptera Vespidae): a first assessment two years after its arrival in Italy. Redia. 2014;97: 189–194.

[46] Goldarazena A, de Heredia IP, Romon P, Iturrondobeitia JC, Gonzalez M, Lopez S. Spread of the yellow-legged hornet Vespa velutina nigrithorax du Buysson (Hymenoptera: Vespidae) across Northern Spain. EPPO Bull. 2015;45: 133–138. doi: 10.1111/epp.12185

[47] Witt R. Erstfund eines Nestes der Asiatischen Hornisse Vespa velutina Lepeletier, 1838 in Deutschland und Details zum Nestbau (Hymenoptera, Vespinae). Ampulex. 2015;7: 42–53.

[48] Bertolino S, Lioy S, Laurino D, Manino A, Porporato M. Spread of the invasive yellow-legged hornet Vespa velutina (Hymenoptera: Vespidae) in Italy. Appl Entomol Zool. 2016; 1–9. doi: 10.1007/s13355-015-0386-z26869722

[49] Keeling MJ, Franklin DN, Datta S, Brown MA, Budge GE. Predicting the spread of the Asian hornet (Vespa velutina) following its incursion into Great Britain. Sci Rep. 2017;7: 6240 doi: 10.1038/s41598-017-06212-0 28740240 10.1038/s41598-017-06212-0 PMC5524706

[50] Villemant C, Barbet-Massin M, Perrard A, Muller F, Gargominy O, Jiguet F, et al Predicting the invasion risk by the alien bee-hawking Yellow-legged hornet Vespa velutina nigrithorax across Europe and other continents with niche models. Biol Conserv. 2011;144: 2142–2150. doi: 10.1016/j.biocon.2011.04.009

[51] Rome Q, Dambrine L, Onate C, Muller F, Villemant C, Garcia Perez A, et al Spread of the invasive hornet Vespa velutina Lepeletier, 1836, in Europe in 2012 (Hym., Vespidae). Bull Société Entomol Fr. 2013;118: 21–22.

[52] Barbet-Massin M, Rome Q, Muller F, Perrard A, Villemant C, Jiguet F. Climate change increases the risk of invasion by the Yellow-legged hornet. Biol Conserv. 2013;157: 4–10. doi: 10.1016/j.biocon.2012.09.015

[53] Hijmans RJ, Cameron SE, Parra JL, Jones PG, Jarvis A. Very high resolution interpolated climate surfaces for global land areas. Int J Climatol. 2005;25: 1965–1978. doi: 10.1002/joc.1276

[54] Warren DL, Glor RE, Turelli M. Environmental Niche Equivalency Versus Conservatism: Quantitative Approaches to Niche Evolution. Evolution. 2008;62: 2868–2883. doi: 10.1111/j.1558-5646.2008.00482.x 18752605 10.1111/j.1558-5646.2008.00482.x

[55] Broennimann O, Fitzpatrick MC, Pearman PB, Petitpierre B, Pellissier L, Yoccoz NG, et al Measuring ecological niche overlap from occurrence and spatial environmental data. Glob Ecol Biogeogr. 2012;21: 481–497. doi: 10.1111/j.1466-8238.2011.00698.x

[56] Thuiller W, Lafourcade B, Engler R, Araújo MB. BIOMOD–a platform for ensemble forecasting of species distributions. Ecography. 2009;32: 369–373. doi: 10.1111/j.1600-0587.2008.05742.x

[57] R Core Team. R: A Language and Environment for Statistical Computing [Internet]. Vienna, Austria: R Foundation for Statistical Computing; 2015 Available: http://www.R-project.org

[58] Barbet-Massin M, Jiguet F, Albert CH, Thuiller W. Selecting pseudo-absences for species distribution models: how, where and how many? Methods Ecol Evol. 2012;3: 327–338. doi: 10.1111/j.2041-210X.2011.00172.x

[59] Marmion M, Parviainen M, Luoto M, Heikkinen RK, Thuiller W. Evaluation of consensus methods in predictive species distribution modelling. Divers Distrib. 2009;15: 59–69. doi: 10.1111/j.1472-4642.2008.00491.x

[60] Monceau K, Bonnard O, Thiéry D. Vespa velutina: a new invasive predator of honeybees in Europe. J Pest Sci. 2014;87: 1–16. doi: 10.1007/s10340-013-0537-3

[61] Milanesio D, Saccani M, Maggiora R, Laurino D, Porporato M. Recent upgrades of the harmonic radar for the tracking of the Asian yellow-legged hornet. Ecol Evol. 2017;7: 4599–4606. doi: 10.1002/ece3.3053 28690790 10.1002/ece3.3053 PMC5496521

[62] Rodrigues JFM, Coelho MTP, Varela S, Diniz-Filho JAF. Invasion risk of the pond slider turtle is underestimated when niche expansion occurs. Freshw Biol. 2016;61: 1119–1127. doi: 10.1111/fwb.12772

[63] Mouquet N, Lagadeuc Y, Devictor V, Doyen L, Duputie A, Eveillard D, et al REVIEW: Predictive ecology in a changing world. J Appl Ecol. 2015;52: 1293–1310. doi: 10.1111/1365-2664.12482

[64] Sheppard CS, Burns BR, Stanley MC. Predicting plant invasions under climate change: are species distribution models validated by field trials? Glob Change Biol. 2014;20: 2800–2814. doi: 10.1111/gcb.12531 24446429 10.1111/gcb.12531

[65] Bessa AS, Carvalho J, Gomes A, Santarém F. Climate and land-use drivers of invasion: predicting the expansion of Vespa velutina nigrithorax into the Iberian Peninsula. Insect Conserv Divers. 2016;9: 27–37. doi: 10.1111/icad.12140

[66] Barbet-Massin M, Jetz W. A 40-year, continent-wide, multispecies assessment of relevant climate predictors for species distribution modelling. Divers Distrib. 2014;20: 1285–1295. doi: 10.1111/ddi.12229

[67] Jarnevich CS, Stohlgren TJ, Kumar S, Morisette JT, Holcombe TR. Caveats for correlative species distribution modeling. Ecol Inform. 2015;29: 6–15. doi: 10.1016/j.ecoinf.2015.06.007

[68] Broennimann O, Guisan A. Predicting current and future biological invasions: both native and invaded ranges matter. Biol Lett. 2008;4: 585–589. doi: 10.1098/rsbl.2008.0254 18664415 10.1098/rsbl.2008.0254 PMC2610080

[69] Mainali KP, Warren DL, Dhileepan K, McConnachie A, Strathie L, Hassan G, et al Projecting future expansion of invasive species: comparing and improving methodologies for species distribution modeling. Glob Change Biol. 2015;21: 4464–4480. doi: 10.1111/gcb.13038 26185104 10.1111/gcb.13038

[70] Petitpierre B, Kueffer C, Broennimann O, Randin C, Daehler C, Guisan A. Climatic Niche Shifts Are Rare Among Terrestrial Plant Invaders. Science. 2012;335: 1344–1348. doi: 10.1126/science.1215933 22422981 10.1126/science.1215933

[71] Strubbe D, Broennimann O, Chiron F, Matthysen E. Niche conservatism in non-native birds in Europe: niche unfilling rather than niche expansion. Glob Ecol Biogeogr. 2013;22: 962–970. doi: 10.1111/geb.12050

[72] Broennimann O, Treier UA, Müller-Schärer H, Thuiller W, Peterson AT, Guisan A. Evidence of climatic niche shift during biological invasion. Ecol Lett. 2007;10: 701–709. doi: 10.1111/j.1461-0248.2007.01060.x 17594425 10.1111/j.1461-0248.2007.01060.x

[73] Lauzeral C, Leprieur F, Beauchard O, Duron Q, Oberdorff T, Brosse S. Identifying climatic niche shifts using coarse-grained occurrence data: a test with non-native freshwater fish. Glob Ecol Biogeogr. 2011;20: 407–414. doi: 10.1111/j.1466-8238.2010.00611.x

[74] Guisan A, Petitpierre B, Broennimann O, Daehler C, Kueffer C. Unifying niche shift studies: insights from biological invasions. Trends Ecol Evol. 2014;29: 260–269. doi: 10.1016/j.tree.2014.02.009 24656621 10.1016/j.tree.2014.02.009

