# Supplementary material for: Can species distribution models really predict the expansion of invasive species?
Source: PLoS One. 2018 Mar 6;13(3):e0193085. doi: 10.1371/journal.pone.0193085 (PMC5839551; doi:10.1371/journal.pone.0193085)
Supplement: S1 Table — (DOCX) [file pone.0193085.s001.docx]

**S1 Table. Comparing SDMs predictive accuracy for models trained with or without native data.** Results from the paired t-tests between the percentiles of validation points predicted when accounting for both native and invasive data vs. when accounting for invasive data only.

| Cut-off year | t | df | Estimate (mean of the differences) [95% CI] | p-value |
| --- | --- | --- | --- | --- |
| 2006 | 3.9 | 3619 | 1.07 [0.53,1.61] | 1.1*10^-4^ |
| 2007 | -33.5 | 3571 | -6.82 [-7.21, -6.42] | < 10^-10^ |
| 2008 | -35.2 | 3485 | -8.69 [-9.17, -8.21] | < 10^-10^ |
| 2009 | -26.5 | 3156 | -7.63 [-8.20,-7.07] | < 10^-10^ |
| 2010 | -7.1 | 2533 | -2.21 [-2.81, -1.60] | < 10^-10^ |
| 2011 | -16.7 | 1847 | -5.81 [-6.49,-5.13] | < 10^-10^ |
| 2012 | -5.7 | 1283 | -1.96 [-2.62, -1.29] | 1.1*10^-8^ |
| 2013 | 5.7 | 805 | 1.80 [1.19,2.42] | 1.3*10^-8^ |
| 2014 | -2.0 | 278 | -0.84 [-1.65,-0.01] | 4.6*10^-2^ |
