# Supplementary material for: Can species distribution models really predict the expansion of invasive species?
Source: PLoS One. 2018 Mar 6;13(3):e0193085. doi: 10.1371/journal.pone.0193085 (PMC5839551; doi:10.1371/journal.pone.0193085)
Supplement: S1 Fig — (DOCX) [file pone.0193085.s002.docx]

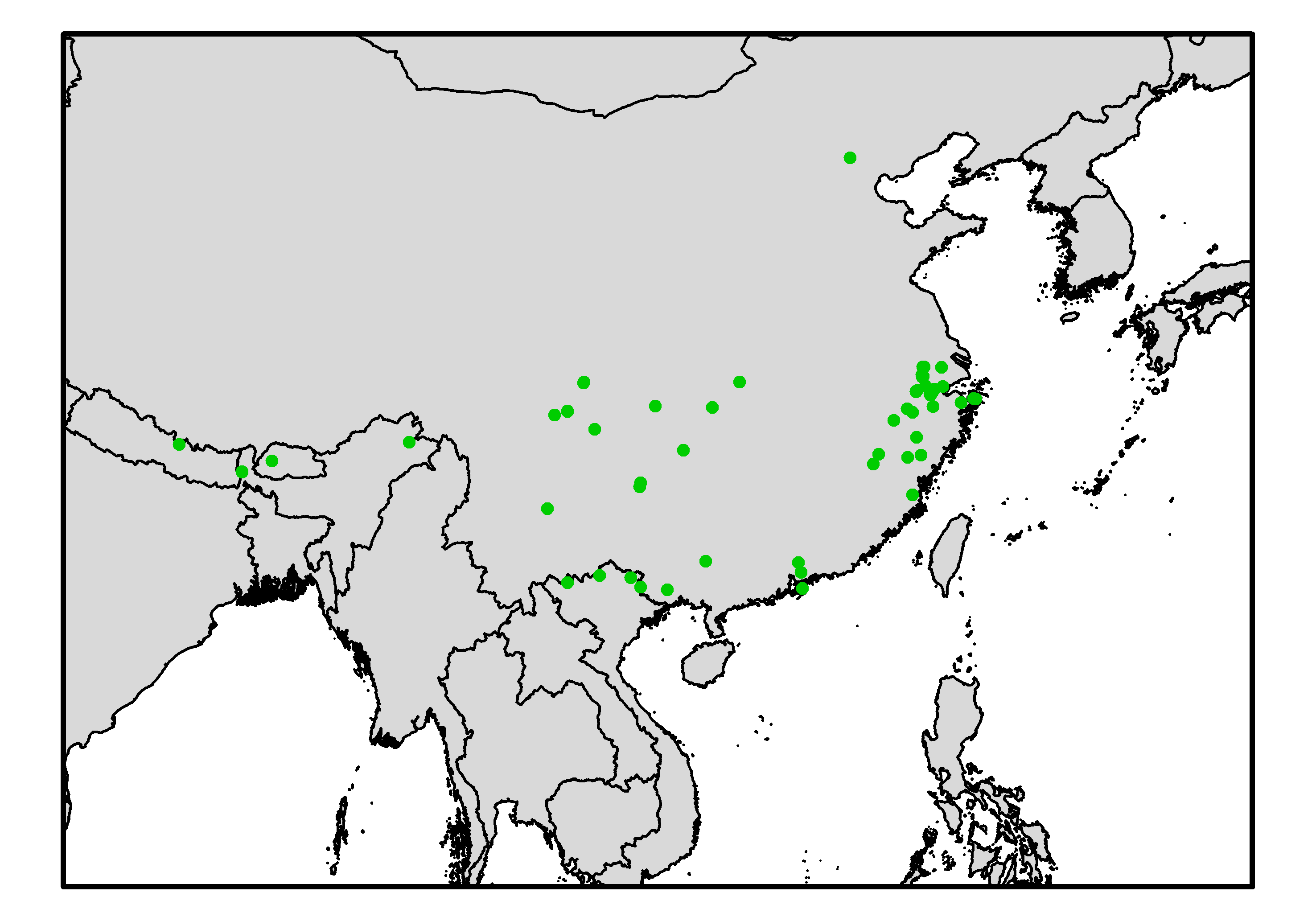


**S1 Fig. Native data.** Localities (n=68) where *Vespa velutina nigrithorax* has been recorded in its native Asian range and used to calibrate the models that accounted for native data (pseudo-absences were drawn from that area).
