# Supplementary material for: Can species distribution models really predict the expansion of invasive species?
Source: PLoS One. 2018 Mar 6;13(3):e0193085. doi: 10.1371/journal.pone.0193085 (PMC5839551; doi:10.1371/journal.pone.0193085)
Supplement: S2 Fig — (DOCX) [file pone.0193085.s003.docx]

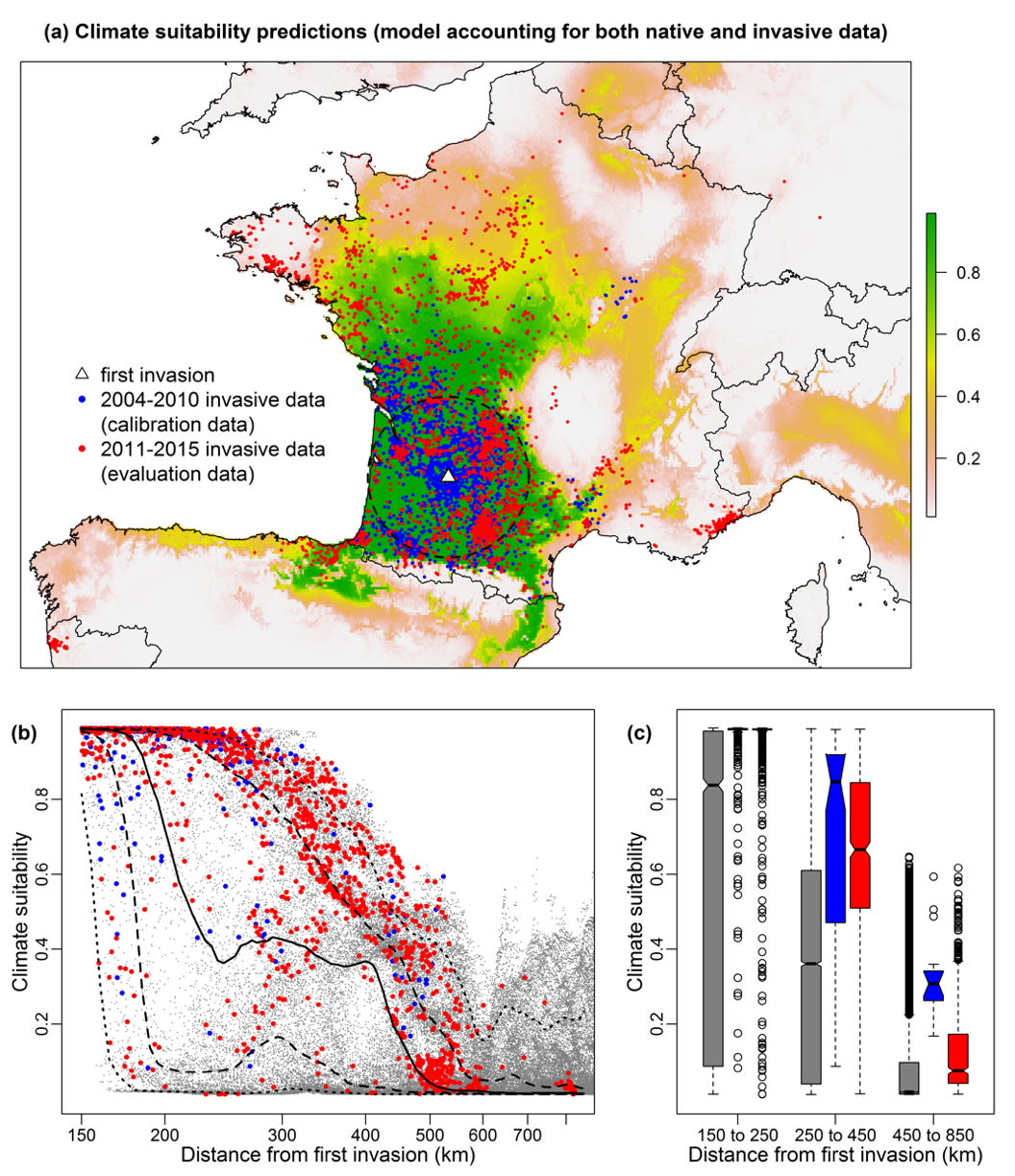


**S2 Fig. SDMs predictions and predictive accuracy accounting for both native data and invasive data.** Similar to Fig 2, apart from climate suitability being predicted by accounting for both native data and invasive data.
