# Supplementary material for: Can species distribution models really predict the expansion of invasive species?
Source: PLoS One. 2018 Mar 6;13(3):e0193085. doi: 10.1371/journal.pone.0193085 (PMC5839551; doi:10.1371/journal.pone.0193085)
Supplement: S3 Fig — (DOCX) [file pone.0193085.s004.docx]

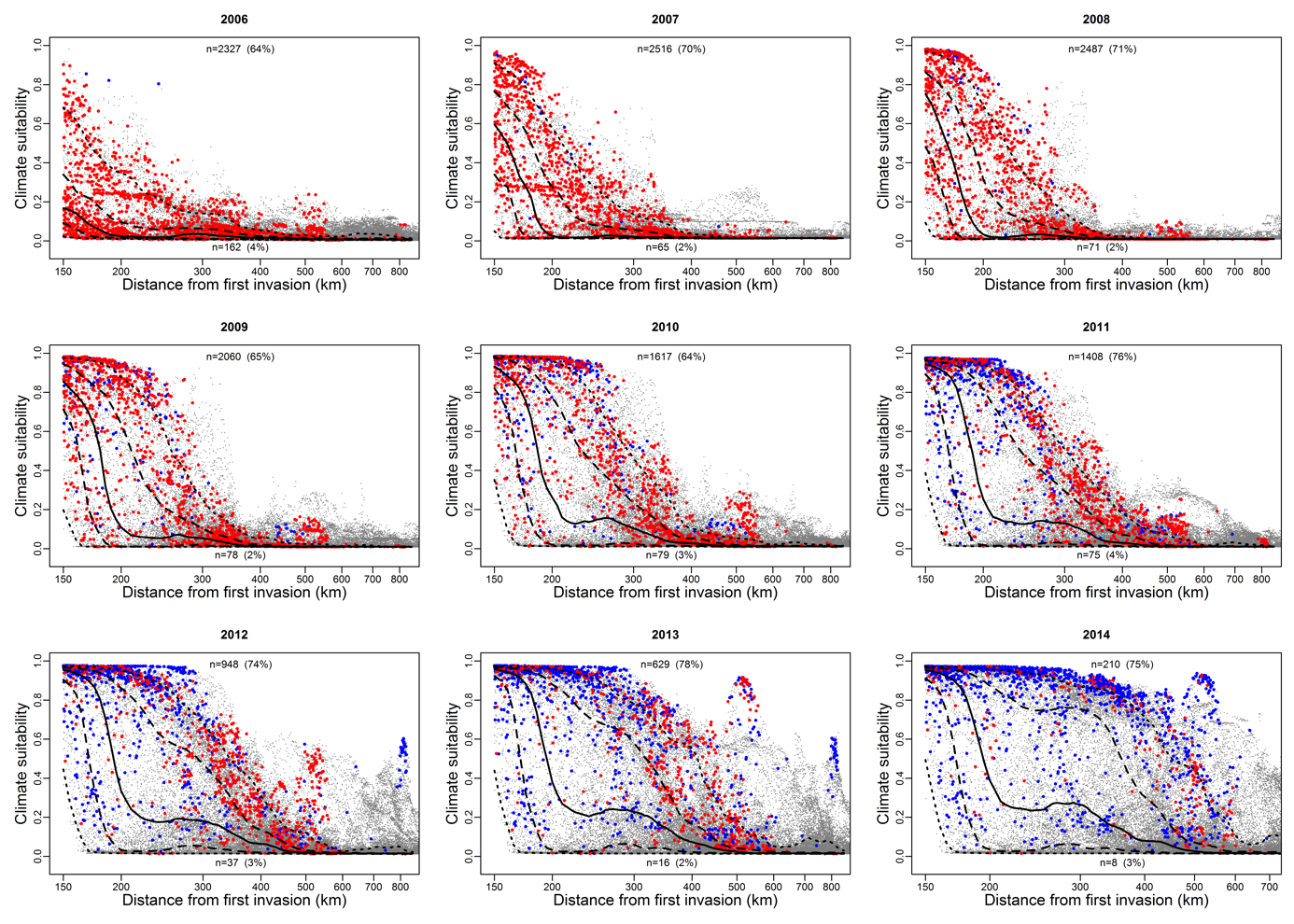


**S3 Fig. SDMs (trained with invasive data only) predictive accuracy for all cutoff years.** Climate suitability (accounting for invasive data only) of all possible points (between 150 and 850 km of the first invasion data) according to their distance to the first invasion (grey points) for all cutoff years (Results similar to Fig. 2b for all different cutoff years). The full line represents the median climate suitability according to the distance, whereas the dotted lines represent the 10 %, 30 %, 70 % and 90 % quantiles (blue and red points represent calibration and evaluation points). Evaluation (red) points above the median have a higher predicted suitability than expected given their distance to the first invasion occurrence. The amount (and percentage) of evaluation points falling above the 70 % quantile (respectively below the 30 % quantile) is specified on top (respectively at the bottom) of the plot.
